# Pathogenesis and Management of Brugada Syndrome: Recent Advances and Protocol for Umbrella Reviews of Meta-Analyses in Major Arrhythmic Events Risk Stratification

**DOI:** 10.3390/jcm11071912

**Published:** 2022-03-30

**Authors:** Hasina Masha Aziz, Michał P. Zarzecki, Sebastian Garcia-Zamora, Min Seo Kim, Piotr Bijak, Gary Tse, Hong-Hee Won, Paweł T. Matusik

**Affiliations:** 1 Faculty of Medicine, Jagiellonian University Medical College, 31-530 Kraków, Poland; masha.aziz@student.uj.edu.pl; 2 Department of Anatomy, Jagiellonian University Medical College, 31-034 Kraków, Poland; michal.zarzecki@uj.edu.pl; 3 Cardiology Department, Delta Clinic, Rosario S2000, Argentina; szamora@sanatoriodelta.com.ar; 4 Samsung Advanced Institute for Health Sciences & Technology (SAIHST), Sungkyunkwan University, Samsung Medical Center, Seoul 06351, Korea; minseolike@korea.ac.kr; 5 John Paul II Hospital, 31-202 Kraków, Poland; p.bijak@szpitaljp2.krakow.pl; 6 Cardiac Electrophysiology Unit, Cardiovascular Analytics Group, Hong Kong, China; gary.tse@kmms.ac.uk; 7 Tianjin Key Laboratory of Ionic-Molecular Function of Cardiovascular Disease, Department of Cardiology, Tianjin Institute of Cardiology, Second Hospital of Tianjin Medical University, Tianjin 300070, China; 8 Kent and Medway Medical School, University of Kent and Canterbury Christ Church University, Canterbury CT2 7FS, UK; 9 Samsung Advanced Institute for Health Sciences & Technology (SAIHST), Samsung Genome Institute, Samsung Medical Center, Seoul 06351, Korea; wonhh@skku.edu; 10 Department of Electrocardiology, Institute of Cardiology, Faculty of Medicine, Jagiellonian University Medical College, 31-202 Kraków, Poland; 11 Department of Electrocardiology, The John Paul II Hospital, 31-202 Kraków, Poland

**Keywords:** Brugada syndrome, pathogenesis, management, primary electrical disease, arrhythmic events, sudden cardiac arrest, sudden cardiac death, risk stratification, review, protocol

## Abstract

Brugada syndrome (BrS) is a primary electrical disease associated with life-threatening arrhythmias. It is estimated to cause at least 20% of sudden cardiac deaths (SCDs) in patients with normal cardiac anatomy. In this review paper, we discuss recent advances in complex BrS pathogenesis, diagnostics, and current standard approaches to major arrhythmic events (MAEs) risk stratification. Additionally, we describe a protocol for umbrella reviews to systematically investigate clinical, electrocardiographic, electrophysiological study, programmed ventricular stimulation, and genetic factors associated with BrS, and the risk of MAEs. Our evaluation will include MAEs such as sustained ventricular tachycardia, ventricular fibrillation, appropriate implantable cardioverter–defibrillator therapy, sudden cardiac arrest, and SCDs from previous meta-analytical studies. The protocol was written following the Preferred Reporting Items for Systematic review and Meta-Analysis Protocols (PRISMA-P) guidelines. We plan to extensively search PubMed, Embase, and Scopus databases for meta-analyses concerning risk-stratification in BrS. Data will be synthesized integratively with transparency and accuracy. Heterogeneity patterns across studies will be reported. The Joanna Briggs Institute (JBI) methodology, A MeaSurement Tool to Assess systematic Reviews 2 (AMSTAR 2), and the Grading of Recommendations, Assessment, Development and Evaluation (GRADE) are planned to be applied for design and execution of our evidence-based research. To the best of our knowledge, these will be the first umbrella reviews to critically evaluate the current state of knowledge in BrS risk stratification for life-threatening ventricular arrhythmias, and will potentially contribute towards evidence-based guidance to enhance clinical decisions.

## 1. Introduction

Brugada syndrome (BrS) is a primary electrical disease associated with arrhythmias and an elevated risk of sudden cardiac death (SCD) [1,2]. It was described by Pedro and Josep Brugada in 1992 as a syndrome comprised of “right bundle branch block, persistent ST segment elevation and SCD” [3]. However, the description of the electrocardiographic (ECG) changes considered today as type 1 BrS ECG pattern was first published in 1953 [4]. The prevalence of individuals with the Brugada ECG patterns differs largely among various regions and populations of the world [5] and is more common than BrS. Pooled worldwide prevalence of BrS is 0.5 per 1000 [6] based on ECG patterns, with highest prevalence in Southeast Asia of 3.7 per 1000, reaching up to 17.7 per 1000 in Thailand [6,7]. BrS is approximately nine times more common in males [8,9], and is one of the leading causes of SCD in males below age 40 in southeast Asia [10]. Patients with BrS are considered symptomatic if they have a history of aborted SCD, ventricular fibrillation (VF), sustained ventricular tachycardia (VT), or syncope [11,12]. 

BrS usually presents during the third or fourth decade of life, and about 63% of patients are asymptomatic at diagnosis [7,11]. However, syncope or major arrhythmic events (MAE) can occur at any age, or SCD may even present as the first event [11]. BrS contributes towards sudden infant death syndrome, SCD in children, and estimated to cause at least 20% of all SCDs in individuals with anatomically normal cardiac structures [9,11,13,14].

## 2. Pathogenesis of BrS

BrS was previously described as an autosomal-dominant inherited disorder with incomplete penetrance, and absent or benign structural heart abnormalities [1,11]. The lack of significant structural heart disease in BrS patients may be visualized by echocardiography, angiography, or ventriculography [14,15]. However, magnetic resonance imaging in subgroups of patients with BrS revealed enlarged right ventricular (RV) volumes, increased RV outflow tract (RVOT) area, or mild RV wall motion abnormalities [16]. The pathomechanism observed in BrS patients involves depolarization and repolarization abnormalities, inflammation of myocytes, and fibrosis in RVOT and/or RV [9,12]. A recent study performed on whole hearts from deceased patients, whose SCD was accounted to BrS, showed biventricular myocardial fibrosis, especially in the epicardium of the RVOT [17]. RV myocardium in a number of patients with BrS type 1 ECG pattern have showed histological changes comparable to arrhythmogenic RV cardiomyopathy (ARVC), and indicate possible autoimmune causes of myocardial inflammation in BrS patients [9].

Genetic etiology, identified in about 14–34% cases, is primarily associated with sodium voltage-gated channel alpha subunit 5 (SCN5A) gene mutation affecting cardiac channels [9,18,19]. SCN5A gene encodes for the α-subunit of the sodium channels in the heart and mutations in the gene lead to reduced expression of Nav1.5 α-subunit proteins, loss of functional sodium channels, and impaired phase 0 action potential [11]. At present, other genes have been identified as susceptibility genes for BrS, and BrS is now considered an oligogenic or polygenic disease [9,20].

Currently, potassium, chloride, and calcium ion channels involved in the cardiac depolarization and repolarization process have also been described as associated with channelopathies caused by the dysfunction of regulatory proteins [21]. For example, excess outflow of potassium current during early repolarization or reduced inward current via calcium channels may contribute to BrS pathophysiology [21]. The reduced inward current flow of sodium in BrS patients may result in prolonged PR (PQ) interval, first degree atrioventricular block [22], slow cardiac conduction (intraventricular and His–Purkinje), phase 2 reentry and premature repolarization [21], low-amplitude and high-frequency electrical activity in RVOT epicardium (late potentials), and ventricular arrhythmias [12,23].

**Figure 1:**
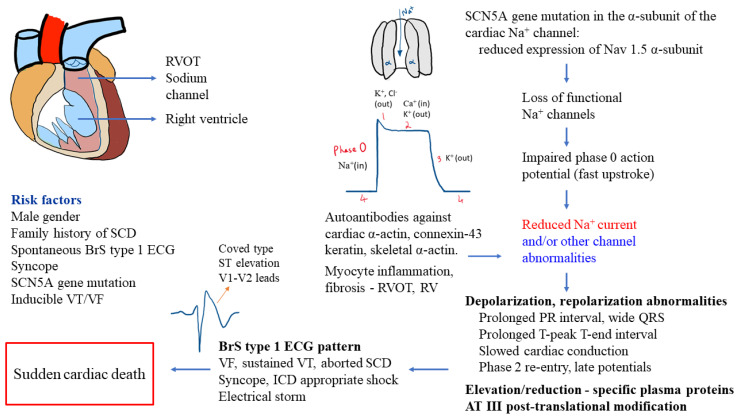
Overview of pathomechanisms involved in Brugada syndrome [9,10,11,12,21,22,24,25,26,27]. Abbreviations: AT—antithrombin; BrS—Brugada syndrome; Ca^2+^—calcium; ECG—electrocardiogram; K^+^—potassium; ICD—implantable cardioverter–defibrillator; Na^+^—sodium; SCD—sudden cardiac death; SCN5A—sodium voltage-gated channel alpha subunit 5; RV—right ventricle; RVOT—right ventricular outflow tract; VF—ventricular fibrillation; VT—ventricular tachycardia.

A recent study identified autoantibodies in the myocardium of BrS patients against cardiac proteins (α-actin, skeletal α-actin, connexin-43, keratin) and observed abnormal expression of Nav1.5 α-subunit proteins [9]. Another study reported distinct elevation (apolipoprotein E, clusterin, prothrombin, vitamin-D-binding protein, complement-factor H, voltage-dependent anion-selective channel protein 3, vitronectin) or reduction (alpha-1-antitrypsin, angiotensinogen, fibrinogen) in plasma proteome of BrS patients and relatives with SCN5A^Q1118X^ gene mutation compared to their healthy family members without the gene mutation, as well as antithrombin-III post-translational modifications [24]. Figure 1 displays an overview of the pathomechanism processes in BrS.

## 3. Diagnostics and Risk Stratification

The definitions of BrS can vary depending on the guidelines used. The European Society of Cardiology (ESC) guidelines proposes that any subject with spontaneous or drug-induced type 1 Brugada ECG pattern be classified as BrS [28]. However, some investigators suggest that this may lead to overdiagnosis, and specific symptoms or clinical data are required to confirm diagnosis [29,30]. Clinical, ECG, and laboratory markers have been found to be useful in diagnostics and risk stratification in diverse groups of patients [31,32,33,34,35,36,37,38,39,40,41]. Despite progress in SCD prevention, the optimal diagnostics and risk stratification in BrS are a major clinical challenge [42,43,44].

### 3.1. Diagnostics in BrS

The ESC definition of BrS diagnosis states that patients must display BrS ECG pattern with ST-segment elevation ≥ 2 mm in at least one lead (V1-V2) placed in the second, third, or fourth intercostal spaces [28]. This may appear spontaneously or following intravenous drug provocation with class Ia (ajmaline or procainamide) or class Ic (flecainide or pilsicainide) sodium channel blockers [28]. The BrS type 1 ECG pattern may also be induced by fever and exercise tests [8,9,10,11,45]. The unique ECG pattern is often short-lasting, and only depending on standard 12-lead ECG may lead to underdiagnosis of 65% patients, especially those who need modified high leads or drug provocation [9]. Therefore, prolonged cardiac monitoring might be highly valuable for diagnostic process [46]. When the Brugada ECG pattern is present without life-threatening arrhythmias or SCD, after exclusion of BrS, it is known as Brugada phenocopy [8].

The Shanghai scoring system for BrS diagnosis is based on ECG, family history, clinical symptoms, and genetics, and assigns a score of ≥3.5 for probable and/or definitive BrS (type 1 BrS ECG pattern–spontaneous or drug-induced), a score from 2 to 3 for possible BrS, and a score of <2 is nondiagnostic [9,47,48]. Additionally, a score of 3 was for fever-induced BrS type 1 ECG, a score of 2 for convertible drug-induced type 2 or 3 BrS ECG pattern, a score of 2 for definite BrS in family (first-/second-degree relative), a score of 0.5 for atrial fibrillation (AF) or atrial flutter in patients younger than age 30 ( no alternative etiology), and a score of 0.5 for probable pathologic genetic mutation which may lead to BrS [9]. SCN5A gene-mutation type and a BrS genetic risk score is associated with BrS phenotype in patients with spontaneous type 1 BrS ECG pattern or family members with SCN5A mutations [49]. Importantly, MRI studies have shown a correlation between maximal ECG ST segment elevation and maximal RVOT area in the presence of BrS type 1 ECG pattern [50].

### 3.2. Risk Stratification in BrS

ESC guidelines on ventricular arrhythmias and the prevention of SCD [28] recommend focusing on risk stratification and clinical decisions in the presence of previous SCA or documented spontaneous sustained VT, spontaneous diagnostic type 1 BrS ECG pattern, syncopal episodes, and inducible VF during programmed ventricular stimulation (PVS) (using two–three extrastimuli at two sites). According to the American Heart Association, the American College of Cardiology, and the Heart Rhythm Society (AHA/ACC/HRS) recommendations, for additional risk stratification in asymptomatic BrS patients and in patients with spontaneous type 1 BrS ECG pattern, electrophysiological study (EPS) with PVS (using single and double extrastimuli) may be beneficial [51]. Wakamiya et al. evaluated the emphasis of arrhythmic syncope history or unexplained syncope and VF inducibility by ≤two extrastimuli during PVS according to new guidelines of the Japanese Circulation Society to help determine ICD indication in patients with BrS [52]. The research studied 234 BrS patients where 20% had VF history, 43% had syncope history, and 37% were asymptomatic at diagnosis [52]. Patients underwent PVS at RV apex or RVOT (1–3 extrastimuli) and mean follow-up was 6.9 ± 5.2 years [52]. The study underlined a less aggressive approach for PVS in BrS risk stratification.

Spontaneous type 1 BrS ECG pattern and syncopal episode history, fragmented QRS (fQRS), and ventricular effective refractory period (VRP) <200 milliseconds have been independently associated with ventricular arrhythmic events in BrS [53]. Moreover, prominent R wave (≥0.3 mV or R/q ≥ 0.75) in lead aVR (aVR sign) was identified to be associated with arrhythmic events in BrS [54]. Fever may precipitate both Brugada type 1 and 2 ECG patterns in patients who have normal baseline ECG [8], and predispose to the development of life-threatening ventricular arrhythmias and SCD [10]. However, a recent study indicated that asymptomatic patients with fever-induced type 1 BrS ECG pattern, negative family history of sudden death, and without spontaneous type 1 BrS ECG pattern are at low risk for future arrhythmic events [55].

Prolonged QRS complex duration, >120 milliseconds on standard 12-lead ECG due to slowed depolarization, was more pronounced in BrS patients expressing symptoms and may predict future MAE [12]. Moreover, T-peak to T-end (Tpe) intervals were identified as novel ECG markers in BrS patients for MAE prediction. High-risk BrS patients had longer Tpe interval in lead V1 and Tpeak-Tend/QT ratio compared to low-risk BrS patients [25]. Recently, based on 12-lead ECG data extracted from automated measurements in BrS patients, novel markers (i.e., ST slope) in predicting arrhythmic events in BrS were identified [27]. The authors stated that, using a weighted scoring system determined from QRS frontal axis, QRS duration, S wave duration and ST slope in lead I, as well as R wave duration in lead III, spontaneous VT/VF incidence may be predicted [27].

A new research performed on patients with drug-induced type 1 BrS ECG pattern who underwent PVS showed that a novel ECG marker dST-Tiso interval (the longest interval from V1-2 in the second, third, or fourth intercostal space) following ajmaline injection to be a significant predictor for the inducibility of ventricular arrhythmias (sustained or hemodynamically significant polymorphic VT of VF requiring direct current shock) [56]. The dST-Tiso interval lies in between the initiation and termination (at the isoelectric line) of the elevated coved ST-segment [56]. The dST-Tiso interval displayed adjusted OR 1.03 (95% CI: 1.01–1.04, *p* < 0.001) for ventricular arrhythmias inducibility. At the same time, dST-Tiso interval > 300 ms displayed 92.0% sensitivity, 90.2% specificity, 82.1% positive predictive value, and 95.8% negative predictive value for VT/VF inducibility prediction [56].

SCN5A gene variants may be important predictors of fatal events in BrS and valuable in risk stratification [57]. Loss-of-function SCN5A mutations have shown association with prolonged ECG conduction parameters (P wave or QRS durations) and increased occurrence of lethal arrhythmic events compared to the non-loss-of-function SCN5A mutations or SCN5A(−) BrS patients [57].

In BrS individuals, the presence of structural anomalies in the epicardium of the RVOT may contribute to arrhythmias [38]. Endocardial unipolar electroanatomical mapping technology may identify RVOT electrical abnormalities with VF inducibility during PVS and assist in BrS risk stratification [38,58]. Endocardial high-density electroanatomical mapping may permit BrS risk stratification in asymptomatic patients (referred for PVS) [38].

Published studies have discussed clinical risk score models in patients with BrS and were reviewed recently in detail [59]. Briefly, the Shanghai Brugada scoring system was predictive for malignant arrhythmic events among patients evaluated for BrS who were asymptomatic (*n* = 271), experienced syncopal episodes (*n* = 99), or had previous VF (*n* = 23) [47]. Importantly, there were no malignant arrhythmic events in patients with a score of 3 or less (possible or nondiagnostic BrS) [47]. In a multicentric study of 1613 BrS patients, 20% symptomatic (after aborted SCA or syncope) at diagnosis, researchers evaluated the Shanghai score of all patients and Sieira score of 461 patients (mean follow-up 6.5 ± 4.7 years) [60]. While both scoring systems identified arrhythmic events risk in patients with significantly elevated or reduced scores, risk stratification was challenging in intermediate-risk patients, e.g., Sieira score 2–4 [60]. Another multicenter international study of 1110 BrS patients developed a risk-score model for SCD or ventricular arrhythmias, and studied 16 proposed ECG/clinical markers for risk stratification and ICD therapy indication [61]. In a median follow-up of 5.33 years, 10.3% of patients had SCD or ventricular arrhythmias, and increased risk was associated with four factors: spontaneous type-1 BrS ECG pattern (14 points), possible arrhythmic syncope or early repolarization in peripheral leads (each 12 points), and type-1 BrS ECG pattern in peripheral leads (9 points) [61].

## 4. Treatment of Patients with BrS

According to the ESC guidelines, implantable cardioverter–defibrillator (ICD) placement is recommended for the management of BrS patients with aborted SCD or documented spontaneous sustained VT, and should be considered in patients with spontaneous BrS type 1 ECG and previous syncope history [28]. Decision on ICD implantation and approaches for the timeline of treatment in BrS patients should involve evaluation of the risk of ventricular arrhythmias, possible complications and adverse events such as inappropriate ICD shocks, and the impact on the patients’ quality of life [62,63]. Pharmacotherapy with quinidine or isoproterenol should be considered in BrS individuals for the treatment of recurrent VT/VF, such as in electrical storms [28,62]. Quinidine may also be an alternate treatment option for supraventricular arrhythmias or in patients with contraindications to ICD placement [28,62].

A relatively novel and promising approach to treatment in BrS patients is catheter ablation. Epicardial catheter ablation of the RVOT may be considered in patients with repetition of appropriate ICD shocks or previous electrical storms [28,64]. In a study of BrS patients with inducible VT/VF, catheter ablation of the epicardium of RVOT resulted in ventricular arrhythmias that were noninducible in majority of the patients, normalization of the BrS type 1 ECG pattern, and no recurrence of VT/VF in the long term follow-up [65]. In BrS individuals, lifestyle choices such as avoiding consumption of excessive alcohols or heavy meals, avoiding drugs or medications which may induce arrhythmias (http:// www.brugadadrugs.org, 26 March 2022), and immediate antipyretic treatment of fevers are of great clinical value [28].

Some of the published data on MAE risk stratification in BrS seems to be inconclusive or based on limited patient groups. In addition, conflicting evidence is present for the value of PVS during EPS in risk stratification [53,66]. Therefore, we propose a series of extensive umbrella reviews to investigate previous meta-analyses on BrS and evaluate current risk stratification options such as genetic testing, ECG, and PVS for MAE risk stratification, and clinical management guidance.

## 5. Protocol for Umbrella Reviews of Meta-Analyses in MAE Risk Stratification in BrS

**Figure 2:**
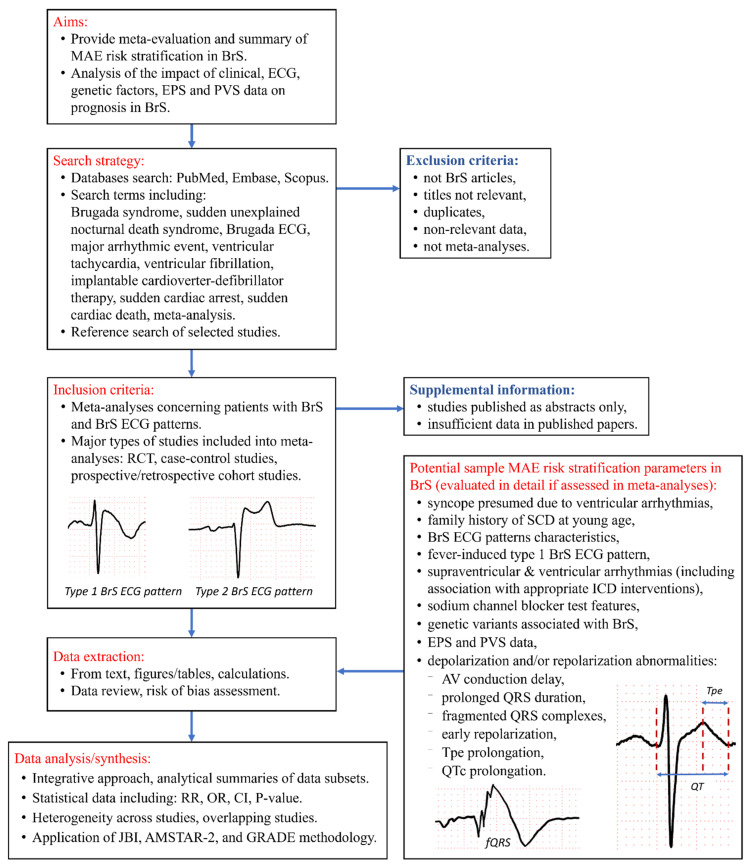
Flow diagram for umbrella reviews on Brugada syndrome risk stratification for major arrhythmic events. Abbreviations: AMSTAR-2—A MeaSurement Tool to Assess systematic Reviews 2; AV—atrioventricular; BrS—Brugada syndrome; CI—confidence interval; ECG—electrocardiograph; EPS—electrophysiology study; fQRS—fragmented QRS; GRADE—Grading of Recommendations, Assessment, Development and Evaluation; ICD—implantable cardioverter defibrillator; JBI—Joanna Briggs Institute; MAE—major arrhythmic events; OR—odds ratio; PVS—programmed ventricular stimulation; QT—QT interval; QTc—corrected QT; RCT—randomized controlled trial; RR—risk ratio; SCD—sudden cardiac death; Tpe—T-peak to T-end interval.

The protocol for our umbrella reviews follows the Preferred Reporting Items for Systematic review and Meta-Analysis Protocols (PRISMA-P) [67] guidelines. Modifications to the protocol will be reported in our final publications. Figure 2 displays an outline of our planned umbrella reviews.

### 5.1. Major Questions of Umbrella Reviews

- What is the association between MAE and clinical factors such as positive family history of SCD in BrS individuals based on an integrative evaluation of previous meta-analyses?
- How can ECG changes such as QRS complexes prolongation, fQRS, AV conduction delay, T-peak to T-end (Tpe) interval, and prolonged QTc interval be used to predict life-threatening ventricular arrhythmias in patients with BrS?
- What role does EPS, PVS, genetic studies, and features of sodium channel blocker challenge have on predicting MAE and sudden fatalities in BrS?

### 5.2. Aims of Umbrella Reviews

The planned umbrella reviews of meta-analyses aim to provide a critical meta-evaluation of MAE risk stratification in BrS, focusing on clinical, ECG, EPS, PVS, and genetic factors. This will significantly advance our understanding of BrS MAE risk stratification and facilitate evidence-based diagnosis and treatment approaches in clinical practice. Our research may potentially alleviate the risk of sudden death and improve the quality of life in BrS patients and their families.

### 5.3. Type and Method of Review

Umbrella review, systematic review, meta-evaluation of meta-analyses.

### 5.4. Search Strategy and Study Selection

Extensive searches of major databases PubMed, Embase, and Scopus will be performed by at least two researchers independently including the following keywords: Brugada syndrome; sudden unexpected nocturnal death syndrome; Brugada ECG; Brugada electrocardiographic; major arrhythmic event; ventricular tachycardia; ventricular fibrillation; appropriate implantable cardioverter–defibrillator therapy; sudden cardiac arrest; sudden cardiac death; prognosis; risk; and meta-analysis. Initial search queries for the databases are shown in Supplementary Materials. Timeline of the search will be until 31 March 2022, and no restrictions will be applied based on language or study publication year. From the search results, after initial screening of titles and abstracts, meta-analyses concerning BrS patients and their relevant outcomes (i.e., MAE—including SCA and SCD) will be considered for our analysis. Reference search of selected studies will be performed to identify additional meta-analyses on BrS and patients with BrS ECG patterns. Relevant abstracts (of nonpublished articles) will be entered into a supplemental table for further review and discussion.

We will evaluate the quality of obtained publications using A MeaSurement Tool to Assess systematic Reviews 2 (AMSTAR 2) [68]. It includes 16 questions to critically appraise and score studies on a scale from high to critically low. Seven domains (2,4,7,9,11,13,15) are most crucial for a systematic review. Two authors will independently apply AMSTAR 2 for each meta-analysis, and variance in evaluation will be resolved by discussion and final consensus among authors.

### 5.5. Inclusion Criteria

Meta-analyses on BrS patients concerning MAE, including sustained VT, VF, appropriate ICD therapy (antitachycardia pacing or shock), SCA, and SCD [28,69] risk stratification. Preferentially, the European Society of Cardiology (ESC) definition of BrS diagnosis will be used [28]. However, to ensure the comprehensiveness of our study, other BrS definitions used before the publication of 2015 ESC guidelines will be included, especially in terms of earlier meta-analyses. Additionally, studies on patients with BrS ECG patterns will be analyzed and considered for inclusion [70].

### 5.6. Exclusion Criteria

We will exclude studies with nonrelevant data, non-BrS articles, and publications that are not meta-analyses (e.g., case reports), unless required for statistical evaluation. During our search process, if we encounter abstracts with relevant meta-analytic data for BrS, and the full-text article of this work was not published, we will add the data into a supplemental table for review as it may be of clinical importance. To avoid duplication of the data, we will exclude:

- older versions of updated meta-analyses;
- publications which included smaller number of studies or smaller number of patients on the same risk stratification tool [71].

### 5.7. Participants/Population

Patients diagnosed with BrS, patients with BrS ECG patterns, and controls (if applicable).

### 5.8. Types of Interventions or Exposures

Our objective is to use an integrative approach to evaluate quantitative data from selected meta-analytic studies in BrS patients (Figure 2):

- clinical factors such as fever-induced BrS ECG pattern, syncope, and positive SCD family history;
- spontaneous and drug-induced type-1 BrS ECG pattern;
- supraventricular and ventricular arrhythmias—including appropriate ICD therapy,
- depolarization and/or repolarization abnormalities due to factors such as atrioventricular conduction delay, prolonged QRS duration, fQRS, early repolarization, late potentials, and Tpe interval prolongation;
- abnormal EPS and/or PVS;
- genetic variants associated with BrS.

### 5.9. Planned Umbrella Reviews Include

- Analysis of clinical (i.e., previous syncope) and ECG factors, including ECG parameters such as prolonged QRS duration, atrioventricular conduction delay, Tpe, fQRS, QTc prolongation, late potentials, arrhythmias, fever-induced type 1 BrS ECG pattern, and the impact of sodium channel blocker challenge features for MAE risk stratification in BrS.
- Evaluation of the influence of genetic factors, and family history of BrS or SCD at young age, on life-threatening ventricular arrhythmias.
- Analysis of EPS and PVS for MAE risk stratification in BrS.

### 5.10. Context

Our umbrella reviews will seek to investigate the impact of clinical factors, data from additional diagnostics or testing, and exposures experienced in BrS patients. We will analyze potential preventive measures, treatments, and the risk of MAE in patients with BrS and BrS ECG patterns. There are no restrictions to context. However, due to the extent of the context, we will consider writing more than one umbrella review on this topic.

### 5.11. Types of Studies

Studies considered for evaluation include meta-analyses of all study designs such as randomized controlled trials, prospective and retrospective cohort studies, and case-control studies.

### 5.12. Condition or Domain Being Studied

Clinical and ECG factors, genetic testing, family history of SCD or BrS, and EPS/PVS for MAE risk stratification or assessment in BrS patients and their families.

### 5.13. Main Outcomes

- Evaluation of association between multiple potential MAE risk stratification parameters and MAE in individuals with BrS (e.g., listed in Figure 2).
- Application of meta-evaluation results to facilitate evidence-based diagnosis and treatment approaches in order to mitigate the risk of SCD and improve the quality of life in BrS patients and their relatives.

### 5.14. Planned Measures of Effect

For each clinical and ECG factors, genetic testing, and EPS/PVS reviewed across meta-analytic studies, we plan to evaluate and compare available data for the relative risk (RR), odds ratio (OR), mean difference (MD), weighted mean difference (WMD), and risk difference (RD), as applicable. Statistical significance will be analyzed in terms of *p*-value <0.05 and 95% confidence intervals (CI). We plan to assess heterogeneity by analyzing Q statistics and I^2^ values reported in the studies.

### 5.15. Data Extraction (Selection and Coding)

Full-text articles and abstracts retrieved from the search strategy and additional sources will be screened based on the eligibility criteria specified above. Data from each study will be extracted into a standardized data extraction template which will include first author name, publication year, inclusion criteria, type of studies, number of studies, databases searched, time period of the search, number of patients included, follow-up information, patient characteristics, and major research findings. Calculations and data extraction from figures and tables will be performed as needed to obtain the data of interest from each study. A comprehensive data review by at least two authors will ensure data quality and completeness of the systematic review. Discrepancies and concerns will be resolved through discussions among authors.

### 5.16. Risk of Bias (Quality) Assessment

At least two authors will perform the search procedure, comprehensive data extraction, and review for quality assessment. Discrepancies about quality of the articles or specific data items will be resolved by discussion and consensus between reviewers. Notes regarding all sources of data and any potential inconsistencies will be discussed.

The Grading of Recommendations, Assessment, Development and Evaluation (GRADE) will be used for evaluating the quality of evidence and presenting clinical management suggestions from our selected studies [72]. The evidence quality will be assessed on a scale from high to very low based on the indication of likely effect and the probability of the effect being different [72]. The GRADE specifications provide guidance for forming questions, selecting and scoring data of interest, analyzing evidence, assessing biases, and handling imprecision and inconsistencies in the results [72].

### 5.17. Strategy for Data Analysis/Synthesis

We will incorporate the Joanna Briggs Institute (JBI) Critical Appraisal Checklist for Systematic Reviews and Research Syntheses for our umbrella reviews [73]. Data will be synthesized using an integrative approach from each study for each category to provide evidence-based analysis for usage in clinical practice.

Data will be reported with transparency, accuracy, and completeness in tabular format. All sources of evidence and calculations will be reported with precision and integrity. Analytic summaries of subsets of data and our research outcome will be described in text, figures, and/or tables. In case of overlapping studies in multiple meta-analyses, inconsistency in data will be reviewed and addressed. The GRADE methodology will be applied for evaluating the quality of the evidence retrieved from our selected studies [72].

For statistical evaluation and comparison of variables from different studies, we plan to include OR, CI, risk ratio, MD, WMD, and RD based on the available data. We will consider reporting frequencies and percentages, and consider the conversion of all the common effect sizes for all factors analyzed to equivalent ORs, if applicable. We plan to determine the heterogeneity across studies according to the variance between the studies (Tau-squared, I^2^). We will consider I^2^ > 50% to reflect significant statistical heterogeneity. For I^2^ < 50%, the fixed-effects model will be used; otherwise, the random-effects model using the inverse variance heterogeneity method will be used. To identify the source of heterogeneity, sensitivity analysis using the leave-one-out method will be considered. To assess possible publication bias, we plan to perform funnel plots, Begg’s, and Egger’s test, if applicable. We plan to calculate sensitivity, specificity, and likelihood ratios of the different risk markers according to the available data. Finally, if the included studies vary substantially in their methodological quality, we will consider a sensitivity analysis including only those studies with a low risk of bias. We plan to use STATA 13 from StataCorp (StataCorp. 2013. Stata Statistical Software: Release 13. College Station, TX, USA: StataCorp LP.) and R software (RStudio Team (2020). RStudio: Integrated Development for R. RStudio, PBC, Boston, MA, USA) for statistical analysis.

## 6. Discussion

Advancement in MAE risk stratification is important for patients with cardiovascular diseases, including arrhythmogenic and conduction disorders and rare arrhythmias [74,75,76,77], which include patients with BrS and their family members. Several problems should be highlighted in diagnosis and risk stratification in patients with BrS. The initial step should be associated with detailed ECG assessment and the exclusion of Brugada phenocopy, where the Brugada ECG pattern is observed during metabolic abnormalities, ischemia, or other causes and is no longer noticed when these conditions resolve [70]. PVS may assist in evaluating the risk of arrhythmias in subgroups of patients such as BrS patients with drug-induced type 1 ECG pattern and experiencing unexplained syncope or patients without symptoms with spontaneous type 1 ECG pattern [78].

Guidelines from both ESC and AHA/ACC/HRS underlined that, at the time of their publication, genetic testing did not influence prognosis in BrS patients [28,51]. However, genetic testing and counseling may be valuable in first-degree relatives of SCD victims as it may help identify inherited BrS [51]. Progress in genetics, including genome-wide association studies and clinical research are promising in BrS patient management. We hope our research outcome will shed new light on potential new or combined factors for SCD risk stratification in BrS, and facilitate future clinical decisions and practice guidelines based on the quality of present evidence.

Our research outcome will hopefully advance our understanding of BrS risk stratification for efficient diagnosis and treatment approaches, and potentially reduce SCD risk by timely interventions such as ICD placement [63] or epicardial catheter ablation of the RVOT [64]. Our umbrella reviews may promote early detection, prevention, and counselling of patients with BrS, who might be susceptible to MAE events triggered by alcohol, fever, heavy meals, specific types of exercise, cocaine, and selected drugs [9,11,45]. Our proposed umbrella reviews will provide a valuable summary of and supplement to meta-analytical research in the scientific community for clinical practice. Further, we may consider specific ethnic and geographical factors for MAE risk stratification in BrS [6]. While highest prevalence of BrS is in Southeast Asia, the prevalence in the United States reaches about 0.012%, and the prevalence in North Africa seems to be the lowest [6,7]. When considering diverse ethnicities, BrS is 9 times more common in Asians than in Caucasians, and 36 times more common in Asians compared to Hispanics based on population-based ECG studies [6].

Our umbrella reviews will pertain to typical limitations of umbrella reviews and will be restricted to analysis of data reviewed and reported by published meta-analyses, and by any limitations of these studies. We would like to invite clinicians and researchers to send us comments on the planned umbrella reviews based on current meta-analyses. We are open to collaboration.

## 7. Conclusions

Our review underlines the complexity of BrS with multiple factors influencing pathogenesis, diagnostics, and risk stratification. To the best of our knowledge, the planned systematic reviews and evaluations of meta-analyses will be the first umbrella reviews to summarize the current state of knowledge in BrS meta-analyses for MAE risk stratification. Our research may contribute valuable evidence-based guidance in clinical decisions, alleviate the burden of SCD, and improve the quality of life in patients with BrS.

## Data Availability

Not applicable.

## Supplementary Materials

The following supporting information can be downloaded at: https://www.mdpi.com/article/10.3390/jcm11071912/s1: Initial search queries for databases.
