# Supplementary material for: Pathogenesis and Management of Brugada Syndrome: Recent Advances and Protocol for Umbrella Reviews of Meta-Analyses in Major Arrhythmic Events Risk Stratification
Source: J Clin Med. 2022 Mar 30;11(7):1912. doi: 10.3390/jcm11071912 (PMC8999897; doi:10.3390/jcm11071912)
Supplement: Supplementary file 1 [file jcm-11-01912-s001.zip › jcm-1608993-supplementary.pdf]

Supplementary Material

Initial search queries for databases:

PubMed:
("Brugada Syndrome"[Mesh] OR “Brugada Syndrome” OR “Brugada's syndrome” OR “Brugada ECG Pattern*” OR “Brugada Type ECG Pattern*” OR “Sudden Unexplained Death Syndrome” OR “Sudden Unexplained Nocturnal Death Syndrome” OR “SUNDS”) AND ("Tachycardia, Ventricular"[Mesh] OR "Ventricular Fibrillation"[Mesh] OR “major arrhythmic event” OR “ventricular tachycardia” OR “ventricular fibrillation” OR “sudden cardiac arrest” OR “SCA” OR “sudden cardiac death” OR “SCD” OR “death” OR “died” OR “mortality” OR “deceased” OR “prognosis” OR “risk” OR “arrhythmia” OR “arrhythmic” OR “outcome” OR “implantable cardioverter-defibrillator therapy” OR "antitachycardia pacing" OR “anti-tachycardia pacing” OR “shock”) AND ("Meta-Analysis" [Publication Type] OR “meta-analysis” OR “meta analysis” OR “meta-analyses” OR “meta analyses”)

EMBASE:
('brugada syndrome'/exp OR 'brugada syndrome' OR ‘Brugadas syndrome’ OR ‘Brugada ECG Pattern*’ OR ‘Brugada Type ECG Pattern*’ OR ‘Sudden Unexplained Death Syndrome’ OR ‘Sudden Unexplained Nocturnal Death Syndrome’ OR ‘SUNDS’) AND ('heart ventricle tachycardia'/exp OR 'heart ventricle fibrillation'/exp OR ‘major arrhythmic event’ OR ‘ventricular tachycardia’ OR ‘ventricular fibrillation’ OR ‘sudden cardiac arrest’ OR ‘SCA’ OR ‘sudden cardiac death’ OR ‘SCD’ OR ‘death’ OR ‘died’ OR ‘mortality’ OR ‘deceased’ OR ‘prognosis’ OR ‘risk’ OR ‘arrhythmia’ OR ‘arrhythmic’ OR ‘outcome’ OR ‘implantable cardioverter-defibrillator therapy’ OR ‘antitachycardia pacing’ OR ‘anti-tachycardia pacing’ OR ‘shock’) AND ('meta analysis'/exp OR ‘meta-analysis’ OR ‘meta analysis’ OR ‘meta-analyses’ OR ‘meta analyses’)

Scopus:
TITLE-ABS-KEY(“Brugada Syndrome” OR “Brugada's syndrome” OR “Brugada ECG Pattern*” OR “Brugada Type ECG Pattern*” OR “Sudden Unexplained Death Syndrome” OR “Sudden Unexplained Nocturnal Death Syndrome” OR “SUNDS”) AND TITLE-ABS-KEY(“major arrhythmic event” OR “ventricular tachycardia” OR “ventricular fibrillation” OR “sudden cardiac arrest” OR “SCA” OR “sudden cardiac death” OR “SCD” OR “death” OR “died” OR “mortality” OR “deceased” OR “prognosis” OR “risk” OR “arrhythmia” OR “arrhythmic” OR “outcome” OR “implantable cardioverter-defibrillator therapy” OR "antitachycardia pacing" OR “anti-tachycardia pacing” OR “shock”) AND TITLE-ABS-KEY(“meta-analysis” OR “meta analysis” OR “meta-analyses” OR “meta analyses”)
